# Quantity Estimation Based on Numerical Cues in the Mealworm Beetle (*Tenebrio molitor*)

**DOI:** 10.3389/fpsyg.2012.00502

**Published:** 2012-11-21

**Authors:** Pau Carazo, Reyes Fernández-Perea, Enrique Font

**Affiliations:** ^1^Edward Grey Institute, Department of Zoology, University of OxfordOxford, UK; ^2^Instituto Cavanilles de Biodiversidad y Biología Evolutiva, Universidad de ValenciaValencia, Spain

**Keywords:** numerical cognition, quantity estimation, sperm competition, numerosity discrimination, sperm competition risk, mate guarding

## Abstract

In this study, we used a biologically relevant experimental procedure to ask whether mealworm beetles (*Tenebrio molitor*) are spontaneously capable of assessing quantities based on numerical cues. Like other insect species, mealworm beetles adjust their reproductive behavior (i.e., investment in mate guarding) according to the perceived risk of sperm competition (i.e., probability that a female will mate with another male). To test whether males have the ability to estimate numerosity based on numerical cues, we staged matings between virgin females and virgin males in which we varied the number of rival males the experimental male had access to immediately preceding mating as a cue to sperm competition risk (from 1 to 4). Rival males were presented sequentially, and we controlled for continuous cues by ensuring that males in all treatments were exposed to the same amount of male–male contact. Males exhibited a marked increase in the time they devoted to mate guarding in response to an increase in the number of different rival males they were exposed to. Since males could not rely on continuous cues we conclude that they kept a running tally of the number of individuals they encountered serially, which meets the requirements of the basic ordinality and cardinality principles of proto-counting. Our results thus offer good evidence of “true” numerosity estimation or quantity estimation and, along with recent studies in honey-bees, suggest that vertebrates and invertebrates share similar core systems of non-verbal numerical representation.

## Introduction

Understanding the evolution of numerical abilities is a mayor challenge in the study of cognition (Shettleworth, 2010). Reports accumulated during the last few years suggest that human and non-human animals share the ability for quantity estimation, which is believed to be the cognitive foundation of higher numerical skills (Dehaene et al., 1998; Hauser, 2000; Feigenson et al., 2004; Hauser and Spelke, 2004; Beran, 2008; Shettleworth, 2010). Quantity estimation has been reported in every mayor group of vertebrates excepting reptiles (mammals, Beran et al., 2008; birds, Rugani et al., 2009; amphibians, Uller et al., 2003; Krusche et al., 2010; fish, Agrillo et al., 2009); and in a few invertebrates (ants, Reznikova and Ryabko, 2011; bees, Dacke and Srinivasan, 2008; beetles, Carazo et al., 2009).

Despite these advances, it is yet unclear whether quantity estimation in non-human animals is based on the same cognitive system as in humans and, if so, how evolutionary ancient this system might be. Part of the problem lies in the difficulty of establishing whether quantity estimation in non-human animals is based on a computation of numerosity itself (i.e., quantity estimation *sensu stricto*), or on non-numerical continuous cues that co-vary with numerosity (i.e., amount estimation; Agrillo et al., 2011; Shifferman, 2012). Quantity estimation is often investigated by exploring the capacity of animals to discriminate between two sets of objects differing in numerosity (e.g., Carazo et al., 2009). However, several continuous features will co-vary with numerosity as more objects are added to a given group of items, such as temporal duration, area, volume, luminance, shape, or perimeter (Agrillo et al., 2009), which may allow for discrimination of numerosity based on non-numerical cues. Therefore, one of the current challenges of research on numerical cognition is hence to understand the relative importance of amount versus quantity-based numerosity mechanisms, particularly in invertebrates, which have so far received considerably less attention than vertebrates in this respect (Menzel et al., 2007; Reznikova and Ryabko, 2011).

A fruitful approach to study cognitive abilities is to stage tasks with ethological validity, where a clear link between cognition and individual fitness can be established (Dukas, 1998; Shettleworth, 2010). As a matter of fact, the field of numerical cognition has experienced a gradual shift from extensive training in captivity or in artificial settings to considering spontaneous expression of numerical competence, and toward understanding how numerical competence functions for particular animals in their natural environments (e.g., Hager and Helfman, 1991; McComb et al., 1994; Uller et al., 2003; Flombaum et al., 2005; Hanus and Call, 2007). Sperm competition provides an ideal context in which to study numerical cognition in many invertebrates (Shifferman, 2012). Sperm competition makes reference to the evolutionary battle of males for the fertilization of a given set of ova (Parker, 1970). A main prediction of sperm competition models is that male allocation of sperm and/or mate guarding should vary according to the probability that a female will re-mate with a different male before laying her eggs (i.e., sperm competition risk; hereafter SCR), and according to the number of males she is expected to mate with (i.e., sperm competition intensity; hereafter SCI). This prediction rests on the assumption that males are somehow able to assess sperm competition levels, which may be accomplished in two ways. First, males may directly determine the risks from past matings by detecting whether a female has recently mated with other males; for example, by assessing the presence of semen in her reproductive tract (e.g., Cook and Gage, 1995; Siva-Jothy and Stutt, 2003). Second, males may assess the future probability that a female will engage in further matings. Several studies with insects have shown that males assess either male density or the operational sex ratio at the time of mating (e.g., Gage, 1991; Simmons, 2001). Both SCR and SCI will co-vary with the number of males present around the time of mating, and males of many species have been show to respond to increasing numbers of rival males by increasing their allocation to sperm competition strategies (e.g., sperm investment and/or mate guarding; Simmons, 2001). Unfortunately, amount and quantity estimation are confounded in most available sperm competition studies, so we know very little about whether quantity estimation in this context relies on numerical or non-numerical cues (reviewed in Shifferman, 2012).

The mealworm beetle (*Tenebrio molitor*) is a highly polygynandrous beetle that has evolved several strategies reflecting an evolutionary history of intense sperm competition (e.g., Happ, 1969; Siva-Jothy et al., 1996; Drnevich et al., 2000; Griffith, 2001; Drnevich, 2003; Carazo et al., 2004). Sperm transfer in this species begins when males fill a pre-formed spermatophore with sperm and transfer it to the female’s bursa during the first 30–60 s of copulation (Gadzama and Happ, 1974). Once inside the female, the spermatophore undergoes a series of eversions before eventually bursting and releasing sperm, about 7–10 min after the end of copulation (Gadzama and Happ, 1974). When a female re-mates with a second male before the sperm from the first male’s spermatophore has been released into the bursa, the second male is capable of preventing sperm release from the first male’s spermatophore (i.e., spermatophore inhibition) and achieves near complete sperm precedence (Drnevich et al., 2000). In response to spermatophore inhibition, males have evolved a short-term anti-aphrodisiac that they transfer to females during mating, and that increases female re-mating intervals by decreasing long-range female attractiveness (Happ, 1969; Griffith, 2001; Seybold and Vanderwel, 2003). However, this anti-aphrodisiac does not prevent re-mating once a female encounters another male, and is probably only effective in avoiding rapid re-mating (<7 min) when male densities are low (Griffith, 2001; Drnevich, 2003). The probability of suffering from spermatophore inhibition is thus likely to be quite low when male densities are low, and males of this species normally devote very little time to mate guarding under such circumstances (Carazo et al., 2004, 2007). However, local populations of *T. molitor* often reach high densities when they colonize pockets of stored grain (Thompson, 1995, 1998), so the risk of spermatophore inhibition is bound to vary considerably depending on varying levels of relative male density at the time of mating. In accordance, males have been shown to respond to high male densities by increasing the amount of time they allocate to guarding their spermatophore (i.e., spermatophore guarding; Carazo et al., 2007). During spermatophore guarding, a male remains in contact with a female, and will actively fight against a rival male attempting to copulate with the guarded female. Despite considerable size differences, spermatophore guarding normally allows males to delay female re-mating sufficiently to enable sperm release into the bursa (Carazo et al., 2007). Hence, short-term mate guarding appears to be an effective mechanisms to prevent spermatophore inhibition, and the fact that its duration depends on existing levels of SCI suggest that males may be capable of assessing the number of rival males present during or immediately preceding mating.

In support of this idea, *T. molitor* males have been shown to be capable of numerosity discrimination, albeit in a different context. Recently, we investigated the existence of quantity discrimination in this species by using a spontaneous two-choice procedure in which males were simultaneously exposed to substrates bearing odors from different numbers of females (≤4). Our results show that *T. molitor* males discriminate between odor sources reflecting different numbers of donor females when given the choice between odors from 1 versus 4 or 1 versus 3 female donors. In particular, and as predicted, males spent more time inspecting sources with odors from more donor females (Carazo et al., 2009). These results suggest that males can discriminate sources of odors reflecting different numerosities with a signature ratio of 1:2, although we were not able to rule out the possibility that males could have been using continuous cues (Carazo et al., 2009).

Our aim here was to test whether *T. molitor* males are capable of estimating numerosity in a different but biologically relevant context in which only numerical cues are available. We designed an experimental setup in which we simulated the situation faced by a male that has to assess the risk of suffering spermatophore inhibition by assessing relative male density (i.e., male-female encounter rate) immediately prior to mating. We staged matings between virgin females and virgin males in which we varied the number of rival males the experimental male had access to immediately preceding matings (i.e., the risk of suffering spermatophore inhibition). We controlled for the temporal duration of male–male contact across treatments, and rival males (1–4) were presented sequentially (and were not present during mating). In these circumstances, experimental males would need to keep a running tally of the number of different rivals encountered before mating in order to gage the risk of spermatophore inhibition (Shifferman, 2012).

## Materials and Methods

All the beetles used in this study originated from stock cultures maintained in our laboratory. These cultures have been running for more than 10 years with regular contributions from other cultures. All growth stages are kept together in plastic trays with a rearing medium consisting of white flour and wheat bran to which chunks of fruit, bread, and various vegetables are added periodically. The culture is covered with filter paper that is sprayed with water for moisture on a daily basis. All containers are kept in well-ventilated, dark storage cabinets, at ambient humidity, and under temperature-controlled conditions.

Subjects used in our experiments were collected from the stock cultures and sexed as pupae by inspection of developing genitalia on the ventral side of the eighth abdominal segment (Bhattacharya et al., 1970). Individuals were examined under a dissecting microscope both as pupae and after eclosion and those with obvious malformations were discarded. Sexed adults of the same age were kept separately in plastic containers measuring approximately 15 cm(height) × 13 cm × 20 cm until used in the experiments. Plastic containers were maintained in the same way as stock cultures. Males and females participating in mating interactions were virgin, sexually mature (i.e., at least 10 days post-eclosion), and never older than 30 days. After staged matings, experimental males were transferred to a plastic container (same conditions as above) and participated in successive trials as introduced rival males (i.e., males introduced to experimental males in the 20 min prior to mating). Trials were conducted at a temperature of 22–25°C, at ambient humidity, and under dim light.

**Figure 1:**
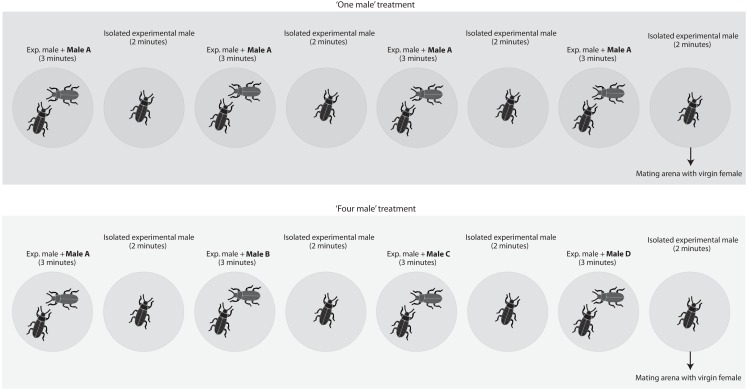
**Two of the four treatments employed (i.e., the “one male” and the “four males” treatments) as an example to illustrate the experimental designed used**. We staged matings between virgin females and virgin males in which we varied the number of rival males the experimental male had access to immediately preceding matings. Each male was introduced in a small 5 cm diameter arena with another male for 3 min and then isolated in a new blank arena for 2 min. We repeated this protocol four times in a row (i.e., overall 20 min) before introducing the experimental male in a mating arena with a virgin female. Each male was subject to one of four different treatments. In the “one male” treatment, the male introduced during the four 3 min periods was always the same. In the “two males” treatment, we used two different males that were alternated. In the “three males” treatment, we introduced three different males and repeated one of the first two males in the last presentation. Finally, in the “four male” treatment each introduced male was different.

To test whether males are capable of estimating numerosity based exclusively on numerical cues, we staged matings between virgin females and virgin males in which we varied the number of rival males the experimental male had access to immediately preceding matings (Figure 1). Twenty minutes before having access to a virgin female, males were subject to the following protocol. Each male was introduced into a small arena (i.e., a 5 cm diameter inverted Petri dish) with another male for 3 min and then isolated in an empty arena for 2 min. We repeated this protocol four times in a row (i.e., overall duration 20 min) before introducing the experimental male into a mating arena with a virgin female. Males were assigned to one of our four treatments: (a) in the “one male” treatment, the male introduced during the four 3 min periods was always the same, (b) in the “two males” treatment, we alternated between two different males (i.e., the same male was never introduced twice in a row), (c) in the “three males” treatment, we introduced three different males in a random order and, in the last 3 min period, we haphazardly selected and introduced one of the first two males again, and (d) in the “four male” treatment, each introduced male was different. We randomized rival male size by randomly selecting males from the sexed cultures. Each of these treatments simulated different average encounter rates with a novel male (i.e., a novel male is encountered once every 20 min in the “one male” treatment, once every 10 min in the “two male” treatment, once every 6.7 min in the “three males” treatment, and once every 5 min in the “four males” treatment). All arenas were clean and free of odors prior to the introduction of “rival” and/or the experimental males. Mating trials begun immediately after the 20 min period in which males were exposed to rival males; i.e., at the end of this period, males were immediately transferred to a mating arena where they had access to a virgin female. If the experimental male failed to initiate courtship within 10 min, the trial was terminated. We used a laptop computer equipped with event-recording software (JWatcher 0.9, Blumstein et al., 2000) to record the duration of the following behaviors:

i) Courtship: begins with the male rapidly tapping the female with its antennae in a rhythmic way. The male then climbs on top of the female making rapid scraping movements with its prothoracic legs against the female’s sides and then proceeds to move its copulatory organ across the female’s rear end until achieving intromission (end of courtship). Tapping with the antennae typically continues through courtship and ends with the onset of copulation.
ii) Copulation: the female lowers her last abdominal sternite and the male introduces the copulatory organ. The pair remains attached by the genitalia for a variable length of time.
iii) Mate guarding: after withdrawing his copulatory organ, the male remains on top of the female and/or dismounts the female and stays immediately adjacent to (i.e., less than 1 cm apart) and usually in direct physical contact with her. Mate guarding typically occurs in bouts that are interrupted by periods in which the members of the pair briefly lose contact with each other. Consequently, the duration of total mate guarding duration is difficult to measure. Our operational measure was restricted to the first bout of mate guarding, which ended when the male and the female were apart from each other (i.e., ca. 1 cm or one body length away from each other) for more than 5 s. Even though this measure is bound to underestimate actual mate guarding, it is an objective conservative measure that correlates strongly with overall mate guarding (Carazo et al., 2007).

Final sample sizes were: “one male” treatment (*n* = 27), “two males” treatment (*n* = 29), “three males” treatment (*n* = 29), and “four males” treatment (*n* = 28). Behavioral data were rank-transformed due to the presence of a few extreme outliers. To look for differences in the time males allocated to courtship, copulation and mate guarding across treatments we performed a robust one-way ANOVA for each of these variables. Significant treatment effects were followed by *post hoc* multiple comparisons using Tukey’s HSD (Quinn and Keough, 2002). As a complementary robust analysis, we winsorized raw data at α = 0.05 to minimize the influence of outliers (i.e., outliers were replaced by the next highest or lowest value, depending on the tail of the distribution), and re-run the one-way ANOVA analyses for all variables. All tests were performed in R v 2.14.0 (R Development Core Team, 2011). All research was conducted in accordance with the animal care and experimentation guidelines provided by the Association for the Study of Animal Behaviour.

## Results

**Figure 2:**
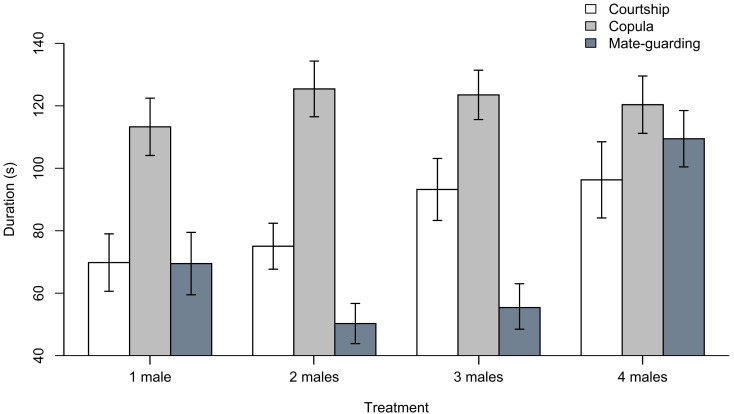
**Duration (in seconds) of courtship, copulation, and mate guarding behaviors in males subject to the “one male,” “two males,” “three males,” and “four males” treatments**. Bar plots show mean ± SEM constructed using data that were previously winsorized at α = 0.05 to minimize the influence of outliers (i.e., outliers were replaced by the next highest or lowest value, depending on the tail of the distribution).

Our ANOVA analyses on ranked data did not detect significant treatment effects for courtship duration (*F*_3_, _109_ = 1.428, *p* = 0.239) or copulation duration (*F*_3_, _109_ = 0.510, *p* = 0.677; Figure 2). ANOVA analyses on winsorized data yielded similar results (i.e., “courtship duration,” *F*_3_, _109_ = 2.011, *p* = 0.117; “copula duration,” *F*_3_, _109_ = 0.328, *p* = 0.805). We did detect a highly significant treatment effect in the time devoted to mate guarding using both rank-transformed (*F*_3_, _109_ = 11.46, *p* < 0.001) and winsorized data (*F*_3_, _109_ = 10.48, *p* < 0.001). In both analyses (for brevity, we report only the ranked data), there was a highly significant difference in mate guarding duration between the “four males” treatment and the “one male” treatment (estimate ± standard error; 29.746 ± 7.810, *t*-value = 3.809, *p* < 0.001), but not between the “two males” (−11.904 ± 7.744, *t*-value = −1.537, *p* = 0.127) or “three males” (−5.559 ± 7.744, *t*-value = −0.718, *p* = 0.474) treatment and the “one male” treatment. Tukey’s HSD test confirmed that the significant treatment effect detected in the ANOVA model was due to the existence of significant differences between the “four males” treatment and the “one male” (difference = 29.746 ± 20.378, *p* = 0.001), “two males” (difference = 41.650 ± 20.017, *p* < 0.001), and “three males” (difference = 35.305 ± 20.016, *p* < 0.001) treatments (again, we found no difference when using winsorized data).

## Discussion

We did not find any evidence that male treatment affected courtship or copula duration (Figure 2). The absence of a treatment effect on courtship duration was expected because it is not involved in spermatophore guarding, and a previous study in this same species reported that courtship duration does not increase with increasing male density (i.e., Carazo et al., 2007). In contrast, in the same study copulation duration was reported to increase in response to increasing male density (as a putative mechanism to extend spermatophore guarding). However, this was in response to higher male densities than those simulated in our experiment (i.e., 10 males; Carazo et al., 2007), which may explain why we did not find an effect on this variable. Finally, we found a highly significant treatment effect on the duration of mate guarding, which is the variable we predicted should be directly affected by increasing male density (i.e., risk of spermatophore inhibition). Our results thus show a marked increase in mate guarding in the “four males” treatment, but we did not detect any significant differences in male behavior when exposed to two or three rival males.

It is important to note that this cannot be taken as evidence that males are not able to discriminate between the “two males” and “three males” treatments and the “one male” treatment. Theory predicts that, in this species, males should increase spermatophore guarding when they perceive a significant increase in the risk of suffering spermatophore inhibition (i.e., the risk that the female they have just mated with will re-mate with another male within the next 7 min, at which time sperm release begins). Female re-mating after sperm release from the first male results in a reduction of approximately 60% in the first male’s paternity share due to sperm dilution by the second male (Drnevich et al., 2000; Drnevich, 2003). However, this outcome is clearly more beneficial than losing all paternity, which may happen if the female re-mates before sperm from the first male is released from the spermatophore. Also, the costs of mate guarding are very high given the mating system of this species, so males cannot prevent females from re-mating with other males before they lay their eggs (i.e., they cannot avoid sperm dilution by other males). In contrast, short-term mate guarding (i.e., spermatophore guarding) is much cheaper and provides males with a tool to avoid spermatophore inhibition (Carazo et al., 2007). For a *T. molitor* male, the crucial question is not whether a female is going to re-mate or not, but whether it is going to do so fast enough so that spermatophore inhibition may take place.

**Figure 3:**
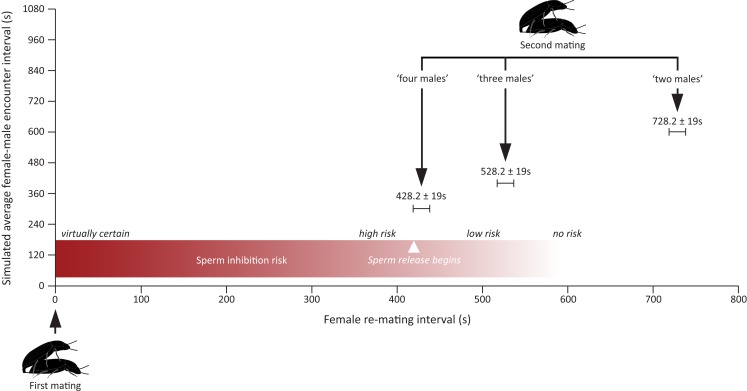
**Figure showing the expected risk of suffering from spermatophore inhibition under three of the four treatments assayed in this study**. Expected female re-mating interval was calculated by summing up the average encounter rate with a new male simulated in each treatment (i.e., a novel male is encountered once every 1200 s in the “one male” treatment, once every 600 s in the “two male” treatment, once every 400 s in the “three males” treatment, and once every 300 s in the “four males” treatment) with the mean duration (mean ± SEM) of mate guarding and courtship taken from the “one male” treatment (i.e., 128.2 ± 19 s). Average courtship and mate guarding duration were calculated from winsorized (α = 0.05) data to prevent overestimation of both parameters due to the presence of outliers (see Materials and Methods). The risk of suffering from spermatophore inhibition is virtually one for re-mating intervals <5 min, high for re-mating intervals around 7 min, and is then bound to drop fast for longer re-mating intervals as sperm release begins (Gadzama and Happ, 1974; Drnevich et al., 2000). This being so, the “four males” treatment is the only one simulating a situation in which males would face a significant increase in the risk of suffering spermatophore inhibition.

It is hence perfectly possible that males in our experiment were able to assess the differences in the number of males in all the treatments, but only responded to the last treatment because it marks the point at which there is a significant increase in the risk of suffering spermatophore inhibition. As a matter of fact, this is exactly what seems to be happening. For sperm inhibition to take place, females have to re-mate with a new male within the first 7 min after the end of their previous mating, at which time sperm release from the first male begins. Given the average encounter rates simulated in our different treatments and the average courtship and mate guarding duration in this species, males should only increase their allocation to mate guarding in response to the last treatment as this is the only treatment in which they face (on average) a risk of loosing their paternity due to sperm inhibition by a second male (Figure 3). Our finding that males only responded to the “four males” treatment hence fits nicely with theoretical expectations.

For the reasons stated above, our results cannot be used to infer information about the operational limit of the cognitive mechanism males are using to assess numerosity (but see Carazo et al., 2009). They do, however, suggest that males of this species possess a sophisticated mechanism that allows them to assess male density, and with it the average risk of spermatophore inhibition that they face after mating with a female. Furthermore, our results suggest that such a mechanisms is probably based on a sequential accumulator model. Given that rival males were presented sequentially to experimental males, the only way for them to assess numerosity is by keeping a running tally of the number of males they encountered during trials. Furthermore, our experimental setup ensured they could only do this by assessing the number of *different* males they encountered because males in all treatments were exposed to the same overall amount of contact with other males. It is also worth noting that the competitive potential of the last male encountered and the average competitive potential of all the males encountered are both expected to be equal across treatments, so this could not explain observed differences in male mate guarding. All in all, these facts make it very unlikely that males could have been using any sort of continuous cue to estimate numerosity.

To conclude, we believe our results offer good evidence of “true” numerosity estimation (i.e., based exclusively on numerical cues) in an insect. Assessment of numerosity in our experimental setup entails a more sophisticated quantity estimation aptitude than mere amount estimation because males need to perform a continuous real-time monitoring of the number of individuals they encounter serially, which meets the requirements of the basic ordinality and cardinality principles of proto-counting (Shifferman, 2012). To our knowledge, in insects such proto-counting ability has only been previously reported conclusively in bees (Dacke and Srinivasan, 2008), although there is some indirect evidence that suggests it may be present in other species (Reinhardt, 2001). In conjunction, these studies suggest that vertebrates and invertebrates share similar non-verbal representational systems allowing quantity estimation based on numerical cues alone. As a corollary, our results also suggest that *T. molitor* males may be capable of individual recognition, a possibility that should be addressed by future studies.

## Conflict of Interest Statement

The authors declare that the research was conducted in the absence of any commercial or financial relationships that could be construed as a potential conflict of interest.
